# Spatial analysis of the epidemiological risk of leprosy in the municipalities of Minas Gerais

**DOI:** 10.1371/journal.pntd.0011381

**Published:** 2023-06-05

**Authors:** Isabela de Caux Bueno, Daniele dos Santos Lages, Francisco Carlos Felix Lana

**Affiliations:** Department of Maternal and Child Nursing and Public Health, Escola de Enfermagem, Universidade Federal de Minas Gerais—UFMG, Belo Horizonte, Minas Gerais, Brazil; Emory University Department of Medicine, UNITED STATES

## Abstract

**Background:**

Leprosy remains a significant public health problem of high importance. This investigation aims to analyze the spatial distribution of the leprosy epidemiological risk in the municipalities of Minas Gerais.

**Methods:**

This ecological study was conducted with new leprosy cases diagnosed from 2004 to 2019 in the municipalities of the state of Minas Gerais. Based on the epidemiological indicators, a composite indicator called the leprosy epidemiological risk index was estimated, classifying municipalities as high, medium, low and very low risk. For the spatial analysis, the global and local spatial autocorrelation statistics were used to identify the spatial distribution of the leprosy epidemiological risk in the periods 2004–2011 and 2012–2019 and classified as High/High, Low/Low, High/Low and Low /High.

**Results:**

Although leprosy is declining in the state of Minas Gerais, the Global Moran Index confirmed the spatial dependence between municipalities for the two analyzed periods, characterizing the formation of clusters. When performing the local spatial autocorrelation, it was found that the macroregions with the highest number of municipalities with high indices, surrounded by other municipalities with high indices (high-high), were Northwest, East, South East, North, and Northeast. The low risk macroregions were Southeast, Center, South-Center and South.

**Conclusion:**

Leprosy has a heterogeneous spatial pattern and remains concentrated in historically endemic areas of the state. It underscores the importance of intensifying actions to combat leprosy in these municipalities and macroregions. Promote improved access to health services and combat stigma and prejudice to eliminate leprosy as a public health problem.

## Author summary

Leprosy remains an important public health problem. More than 200,000 cases are still diagnosed yearly, with Brazil in second place regarding the highest number of cases. Studies involving spatial analysis have been used to assist in planning, implementing, monitoring and evaluating health actions to prioritize areas with higher transmission, therefore, presents a higher risk of illness. Thus, this study aimed to analyze the spatial distribution of the epidemiological risk of leprosy in the municipalities of Minas Gerais. The epidemiological risk was estimated from leprosy indicators related to the magnitude of the endemic disease and the strength of morbidity and transmission of the disease. We identified the formation of areas with higher leprosy epidemiological risk in the state, highlighting the macroregions Northwest, Northeast, East, and South East, which were found to have a higher proportion of municipalities at high epidemiological risk throughout the study period. Moreover, the North macroregion gained epidemiological importance in the last analyzed period. The low risk macroregions were Southeast, Central, Center-South and South. Our results provide potential ways to guide the organization of timely epidemiological surveillance practices and reinforce the importance of intensifying actions to combat leprosy in these municipalities.

## Introduction

Leprosy remains a significant public health problem of high transcendence. Despite showing a decline in the prevalence of the disease globally [1], especially after the introduction of multidrug therapy (MDT), disease control remains a challenge in many places worldwide. In 2019, 202,185 new cases were reported worldwide, with Brazil accounting for 13.8% of these cases (27,863), remaining in second place in the number of absolute cases worldwide and contributing 93% of newly diagnosed cases in America [2]. Moreover, the prevalence of leprosy cases per 10,000 inhabitants has increased in recent years, being 1.09 in 2016 and 1.51 in 2020 [3,4].

The disease remains to be diagnosed in all Brazilian states, highlighting the states of Mato Grosso do Sul, Mato Grosso, Pará, Goiás, and Bahia as they belong to the 26 clusters identified as areas with the higher risk of detecting leprosy [5]. The same study also highlights that the state of Minas Gerais, besides being inserted in three of the 26 clusters identified with a relative risk ranging from 1.9 to 2.2, presented a silent municipality, which did not report any case of the disease, inserted in one of the risk clusters for detecting the disease in the state [5].

Despite the state presenting a decrease in the detection rate of new cases in the general population and children under 15 years of age, there is a persistence of the proportion of grade 2 physical disability in the diagnosis. This suggests the occurrence of late diagnosis and the existence and persistence of sources of infection in the population [6,7]. Moreover, the state of Minas Gerais still presents a heterogeneous distribution of leprosy, and it remains a challenge to control the disease [8].

The monitoring of epidemiological indicators aims to measure the magnitude of the endemic disease and analyze the progress of leprosy elimination as a public health problem [9]. And, to better monitor the epidemiological situation of the disease and understand the differences in the spatial distribution of leprosy, studies involving the geoprocessing technique have been used [10,11]. The investigation of spatial dependence considers the influence of its neighbors, in which events closer to each other tend to be more similar than distant events [12].

This is considered a potential approach as it does not disregard silent areas due to low detection effort, or municipalities with a higher number of cases are favored due to population size and not higher risk [13]. Being an important tool to assist in the planning, implementation, monitoring and evaluation of health actions, giving priority to areas where the transmission is higher [14–16]

This study aimed to analyze the spatial distribution of the leprosy epidemiological risk in the municipalities of Minas Gerais.

## Materials and methods

### Ethics statement

The research was approved by the Research Ethics Committee (REC) of the Federal University of Minas Gerais (UFMG) under opinion n. 490.456. This study was conducted in accordance with Resolution No. 466/2012 of the National Health Council, which establishes guidelines and regulatory standards for research involving human beings. Access to the SINAN database was granted after signing Confidentiality Agreement n. 01/2021 Process n. 1320.01.0029042/2021-12, with the SES/SUBVS-SVE- DVCC-CH, in order to ensure the privacy of the data used.

**Fig 1:**
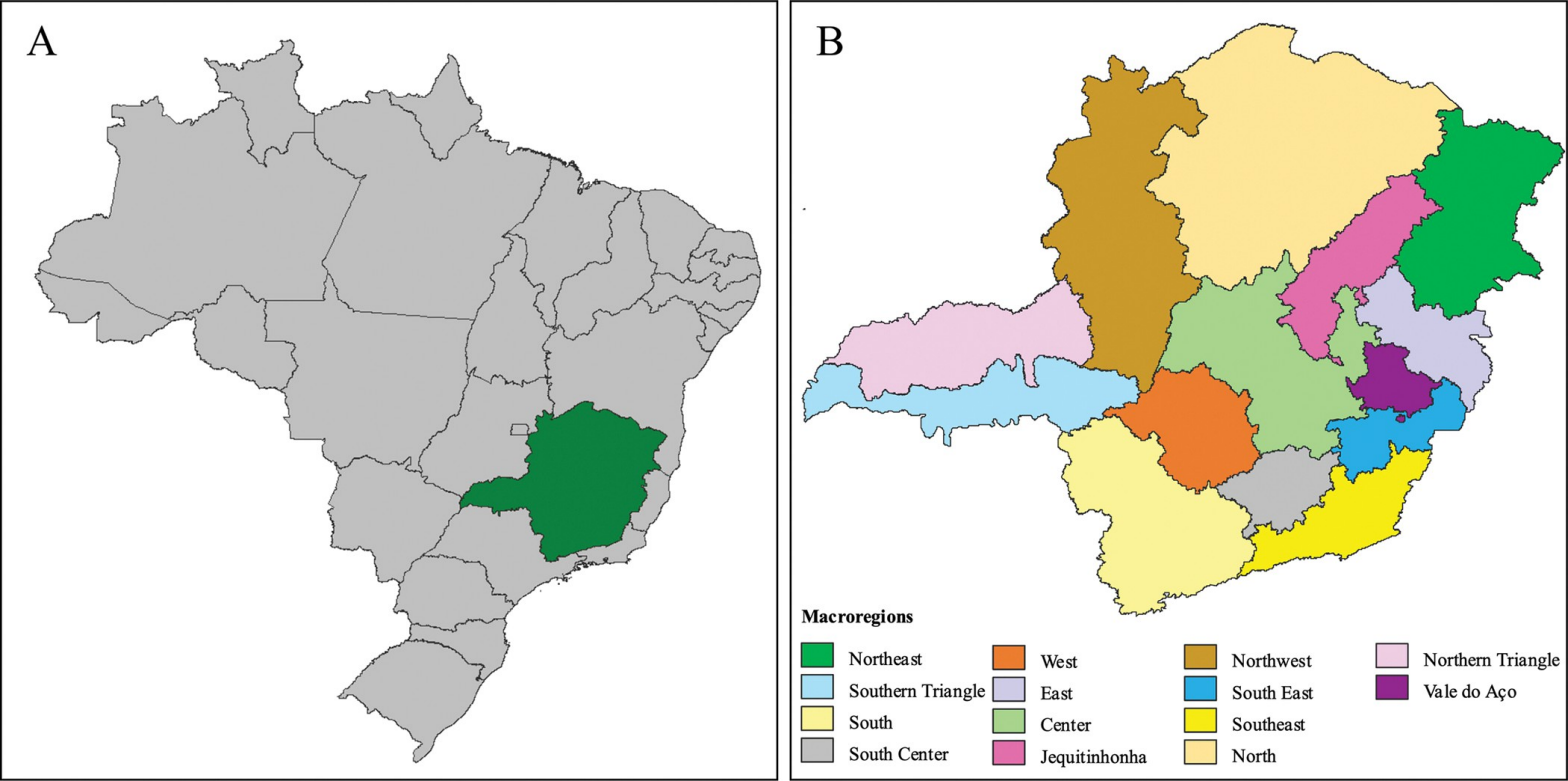
Map of Brazil highlighting the state of Minas Gerais and its divisions by health macroregions. (A) Map of Brazil highlighting the state of Minas Gerais in green. (B) Map of the state of Minas Gerais divided into its health macroregions. The base layer of the map: https://www.ibge.gov.br/geociencias/downloads-geociencias.

This is an ecological study conducted based on the new leprosy cases diagnosed from 2004 to 2019 in the municipalities of the state of Minas Gerais (Fig 1). The period was divided into two sections—from 2004 to 2011 and from 2012 to 2019—considering the long incubation period of leprosy, on average 2 to 7 years [10], and the sensitivity of epidemiological information of the disease to the operational capacity of the services.

The information on new leprosy cases was collected from the SINAN database and made available by the Health Surveillance Undersecretariat of the Minas Gerais State Health Department (SES/MG), referring to consolidated data in March 2021. In order to ensure the privacy of the data used, fully preserve the anonymity and non-stigmatization of patients. The demographic data, referring to the number of inhabitants, was extracted from the IBGE database through the Department of Informatics of the SUS (DATASUS) from the 2010 demographic census and population estimates. The cartographic database in digital and georeferenced format was acquired free of charge from IBGE through its website in the download section.

The data extracted from secondary databases were entered into a database created in Microsoft Excel software (version 2016) for the construction of epidemiological indicators for leprosy: Annual detection rate of new leprosy cases per 100,000 inhabitants; Annual detection rate of new leprosy cases in the population between zero and 14 years old, per 100,000 inhabitants; Rate of new leprosy cases with grade 2 physical disability at diagnosis. The design methodology was conducted following the recommendations of the Brazilian Ministry of Health, described in the "Guidelines for surveillance, attention and elimination of leprosy as a public health problem" [9]. However, it was not possible to calculate the rate of grade 2 physical disability at diagnosis for the year 2007 due to a change in the information system regarding the definition of grade 2 disability. This made the analysis of these data inconsistently for the year 2007 [13,17].

The construction of the Leprosy Epidemiological Risk Index composite indicator was estimated from the abovementioned epidemiological indicators. We used this composite indicator to simplify and synthesize indicators when an overall assessment is needed. The WHO has previously performed this methodological approach of integrated analysis of leprosy indicators to classify countries at risk [3] and it was earlier constructed and validated using the same indicators of this study [18].

The epidemiological risk index was calculated by the municipality from the average of epidemiological indicators for the period 2004 to 2011 and 2012 to 2019, and applied the ratio:

=[(observedvalue/maximumvalue)]=indicatorsscore

Then the indicator scores were summed and divided by three, and transformed into indices:

Index=sumofleprosyindicatorscores/numberofindicators

The index values varied between 0 and 1, considering “best” the lowest value (0) and “worst” the highest value (1). The classification of the values was based on distribution quartiles. Therefore, the distribution of the interquartile range of the composite indicator obtained different parameters in each period analyzed.

For the first period, from 2004 to 2011, the values were classified as very low risk (≤ 0.04), low risk (0.05 to 0.12), medium risk (0.13 to 0.22), and high risk (> 0.22). For the second period, from 2012 to 2019, the values were classified as very low risk (≤ 0.04), low risk (0.05 to 0.08), medium risk (0.09 to 0.18), and high risk (> 0.18).

To minimize the instability of the raw composite indicator and reduce random fluctuations, the smoothing of incidence rates was applied using the local empirical Bayesian method. That includes spatial effects, calculating the estimate locally using municipalities neighboring the municipality where the indicator is to be estimated, and converging toward a local rather than a global mean [19].

In order to verify the global spatial autocorrelation, the global Moran Index was used as a statistical tool, which provides a single measure for the set of all municipalities, characterizing the entire study region. The index ranges from -1 to +1, where values equal to zero indicate the absence of spatial autocorrelation, and values close to + 1 and -1 indicate the existence of positive or negative spatial autocorrelation, respectively [20].

After confirming the global dependence, the local autocorrelation (Local Index of Spatial Association—LISA) was verified using the local Moran index, which allows identifying critical or transition areas and comparing the value of each municipality with neighboring municipalities [20]. Such an index was based on the order two contiguity matrix of the "queen" type. This matrix type defines units with common boundaries or vertices as neighbors.

Based on LISA, municipalities are positioned in the quadrants of Moran’s I scatter plot diagram as follows:

Q1—High/High (positive values, positive means) municipalities with high rates, surrounded by other municipalities also with high rates and that are, therefore, considered of higher priority for intervention;
Q2—Low/Low (negative values, negative means): municipalities with low index value, surrounded by municipalities with low index values of the same index;
Q3—High/Low (positive values, negative means): municipalities with high index values, surrounded by municipalities with low index values of this less index;
Q4—Low/High (negative values, positive means): municipalities with low index values, surrounded by municipalities with high index values of the same index.

The choropleth maps LISA Map and Moran Map were created based on the scatter plot results for Moran Index and LISA. The LISA map is from the local Moran index to identify different patterns of statistical significance. The Moran map is similar to the LISA map, but only considers areas with significant Moran indices (p <0.05). These areas are plotted according to their location on Moran’s I scatter plot (Q1, Q2, Q3, and Q4) [12,20].

## Results

From 2004 to 2019, 26,116 new leprosy cases were reported in the state of Minas Gerais, with an average of 1,632.3 cases per year. The results of the absolute numbers and their respective indicators are presented in Table 1. Of the total, 4.9% (n = 1,290) of the cases were under 15 years old, with an average of 80.6 cases per year. Grade 2 physical disability at diagnosis was present in 10.9% (n = 2,834) of diagnosed individuals.

**Table 1:** Leprosy epidemiological indicators in Minas Gerais from 2004 to 2019.

	*New cases*	*Cases in children under 15*	*Cases with Grade 2 Physical Disability at diagnosis*	*Overall detection rate* ^*1*^	*Under 15s detection rate* ^*1*^	*Rate of Grade 2 Physical Disability at diagnosis* ^*1*^
**2004**	2984	209	267	13,89	2,04	1,94
**2005**	2760	157	281	14,15	2,14	2,02
**2006**	2415	120	254	12,93	2,15	1,64
**2007**	2103	110	254	11,64	1,94	-*
**2008**	1864	79	200	10,71	1,99	1,22
**2009**	1793	85	174	9,74	1,70	1,26
**2010**	1493	52	182	8,60	1,17	1,15
**2011**	1474	61	150	8,38	1,06	0,73
**2012**	1429	54	168	7,93	0,99	0,89
**2013**	1208	51	119	5,90	0,78	0,74
**2014**	1176	54	124	6,28	0,94	0,68
**2015**	1123	45	131	6,27	1,21	0,84
**2016**	1088	55	145	6,19	1,04	0,93
**2017**	1094	58	114	6,41	1,03	0,62
**2018**	1027	59	112	5,27	0,94	0,61
**2019**	1085	41	159	5,04	0,88	0,73
**Total**	**26116**	**1290**	**2834**	**8,70** ^**2**^	**1,38** ^**2**^	**1,11** ^**2**^

1 Rate/100 thousand inhabitants

2 Average years

*Calculation of the year 2007 excluded

The average detection rate in the general population was 8.71 per 100,000 inhabitants, with the highest annual value of 14.15 per 100,000 inhabitants in 2005 and the lowest of 5.04 per 100,000 inhabitants, recorded in 2019. In the period, there was a reduction of 63.7%.

The average detection rate of leprosy in children under 15 years old was 1.38 per 100,000 inhabitants, with the highest rate recorded in 2006 at 2.15 cases per 100,000 inhabitants and the lowest rate reached in 2019 at 0.78 cases per 100,000 inhabitants. The decrease during the study period was 56.9%. Regarding grade 2 physical disability, the average rate in this period was 1.11 per 100,000 inhabitants and showed a reduction of 62.4% in the period.

For the two periods analyzed, 2004 to 2011 and 2012 to 2019, the composite index of the leprosy epidemiological risk was calculated by integrating the epidemiological indicators studied. From this composite indicator, it was possible to perform the distribution and spatial analysis in the two study periods presented in choropleth maps.

**Fig 2:**
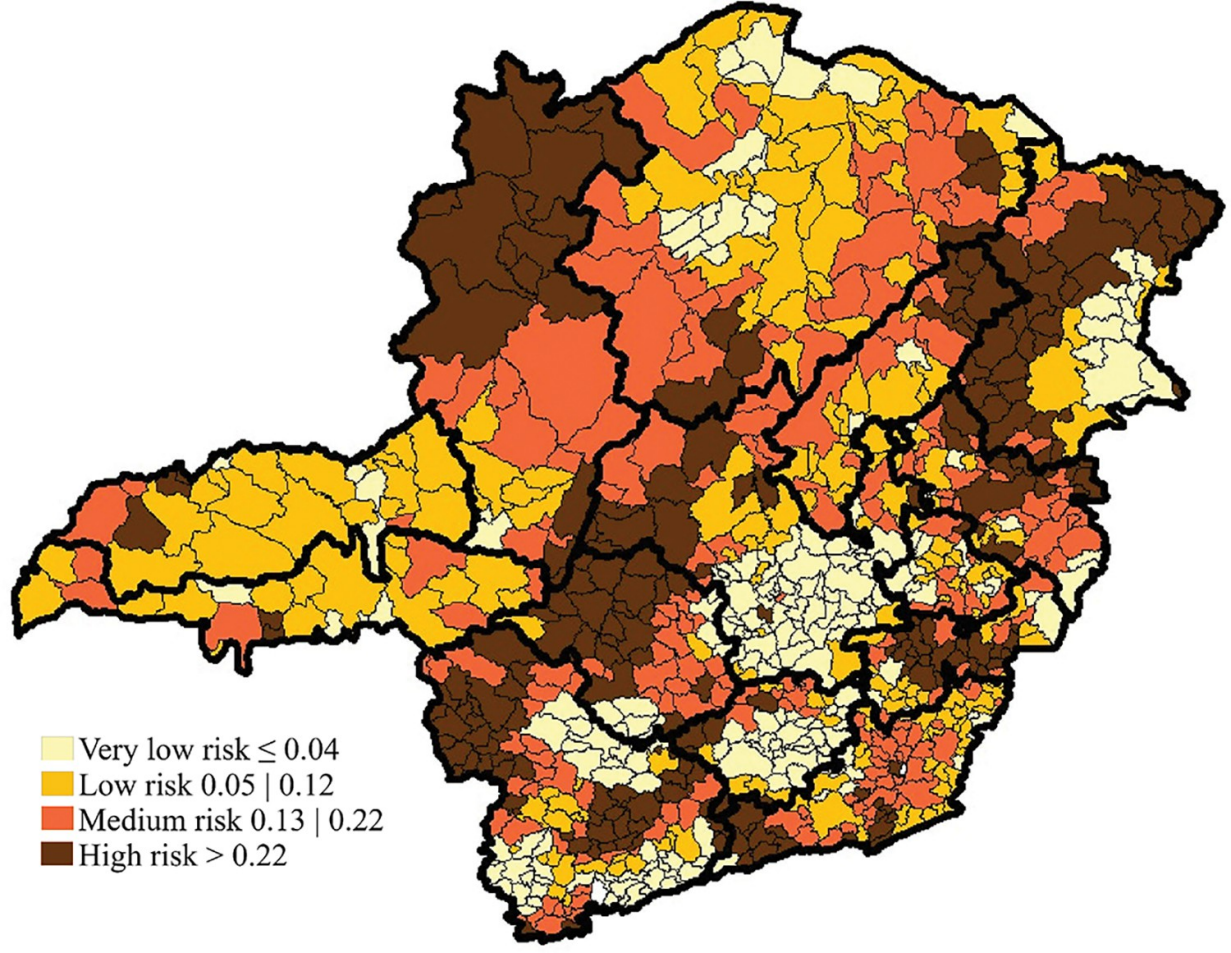
Spatial distribution of the Leprosy Epidemiological Risk index in the municipalities of Minas Gerais from 2004 to 2011. The base layer of the map: https://www.ibge.gov.br/geociencias/downloads-geociencias.

From 2004 to 2011, it is possible to observe that 134 (15.7%) municipalities were classified as very low risk for leprosy, 290 (34%) as low risk, 216 (25.3%) as medium risk, and 213 (25%) as high risk. Fig 2 shows that the municipalities classified as high risk are located mainly in the state’s Northeast, South East, South, West, and Northwest macroregions.

**Fig 3:**
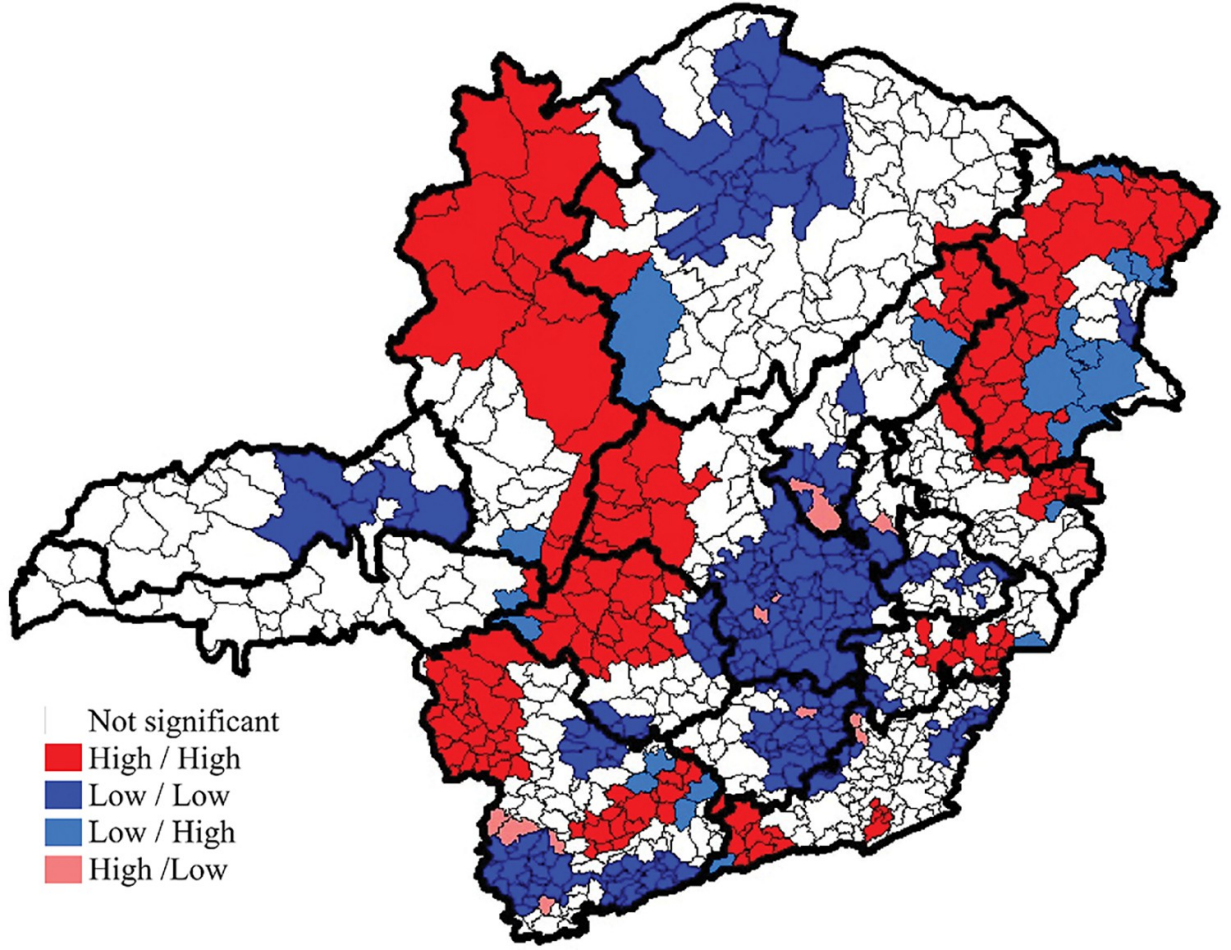
Local spatial autocorrelation of the Leprosy Epidemiological Risk index in the municipalities of Minas Gerais from 2004 to 2011. The base layer of the map: https://www.ibge.gov.br/geociencias/downloads-geociencias.

Once the adjusted leprosy epidemiological risk index was available, the spatial autocorrelation test was applied. Moran’s Global Index confirmed the existence of spatial dependence among the municipalities (0.523; p = 0.001) (Fig 3). This indicates that the analyzed data set is grouped, characterizing the formation of clusters.

When the local spatial autocorrelation was performed using the local Moran index, 48.3% (n = 412) of the municipalities did not show statistical significance. Of the remaining municipalities, 21.5% (n = 183) were classified as high-high, 26.5% (n = 226) as low-low, and few municipalities were classified as low-high (n = 21; 2.5%) and high-low (n = 11; 1.3%).

The macroregions with the highest number of municipalities with high rates, surrounded by other municipalities also with high rates (high-high), were Northwest (94.7%), East (93.3%), Northeast (75.6%), South East (70.0%) and West (66.7%). The macroregions that presented low risk were South Center (91.7%), North (85.7%), Center (83.8%) and South (55.0%).

**Fig 4:**
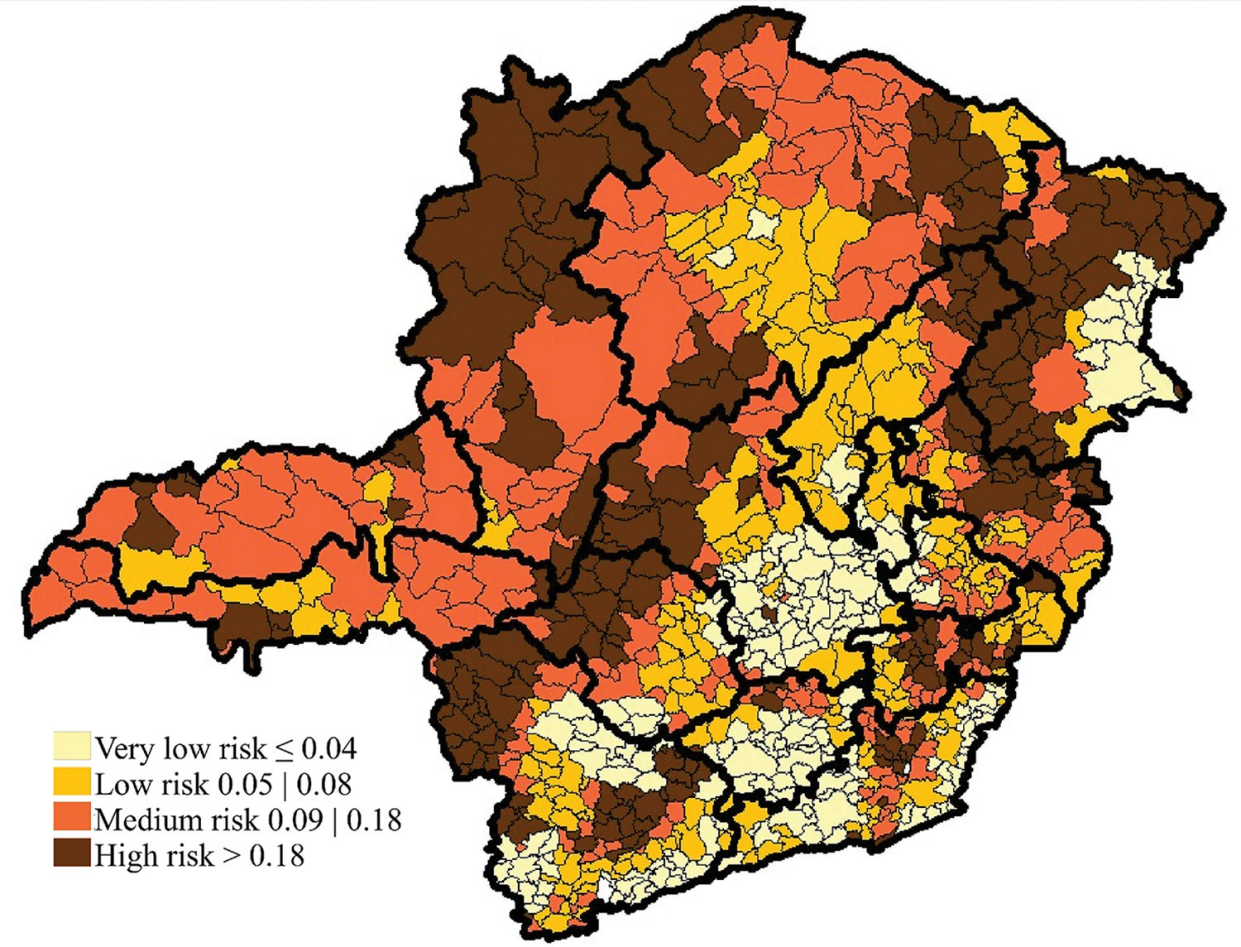
Spatial distribution of the Leprosy Epidemiological Risk index in the municipalities of Minas Gerais from 2012 to 2019. The base layer of the map: https://www.ibge.gov.br/geociencias/downloads-geociencias.

From 2012 to 2019, it was observed that the municipalities classified with high epidemiological risk for leprosy are located mainly in the Northeast and Northwest macroregions. The North region also stands out, which increased the concentration of municipalities with medium and high epidemiological risk from one period to the next (Fig 4).

**Fig 5:**
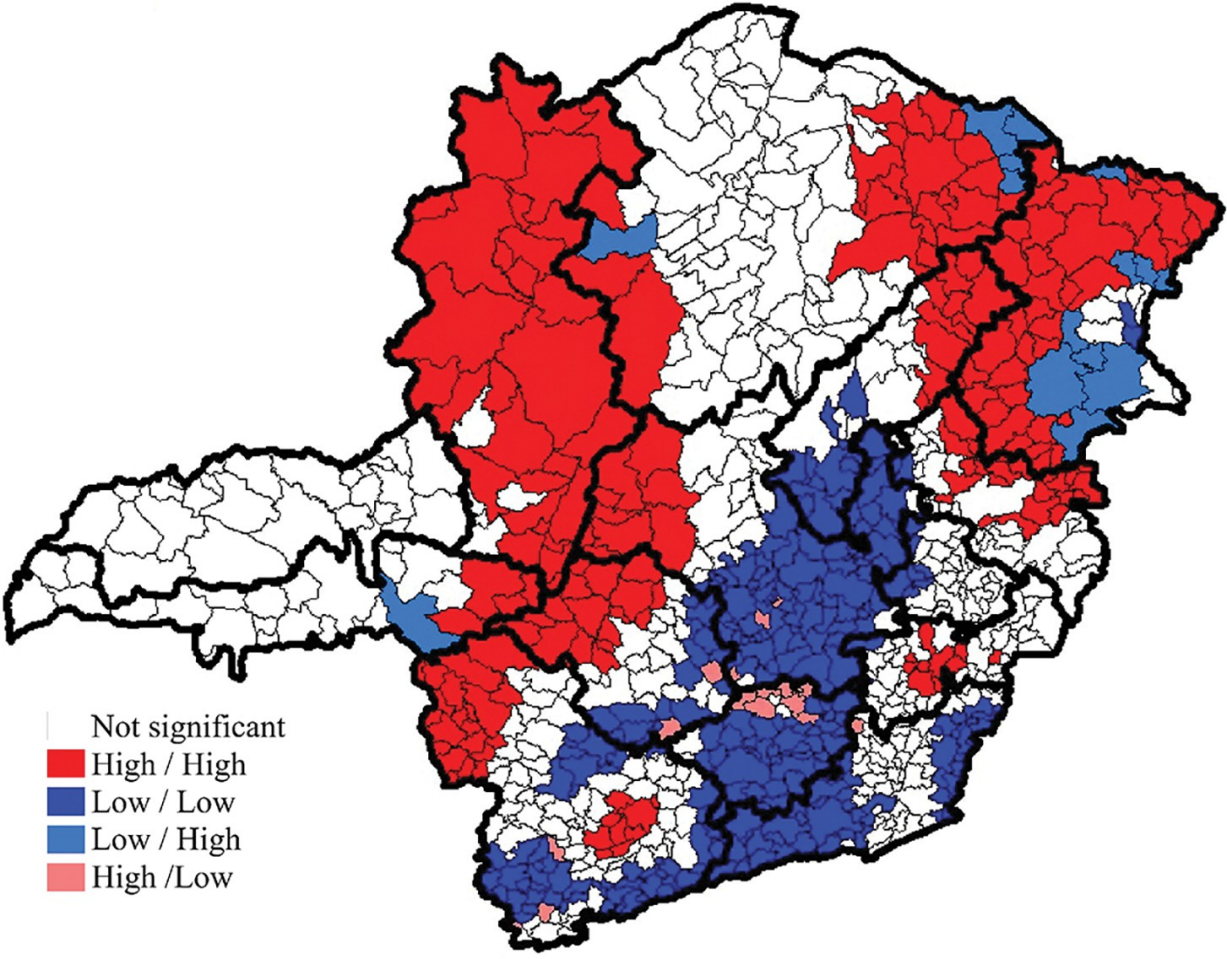
Local spatial autocorrelation of the Leprosy Epidemiological Risk index in the municipalities of Minas Gerais from 2012 to 2019. The base layer of the map: https://www.ibge.gov.br/geociencias/downloads-geociencias.

Moran’s Global Index identified spatial dependence for the adjusted leprosy epidemiological risk index (0.576; p = 0.001). The local spatial autocorrelation identified 44.0% (n = 375) of the municipalities without statistical significance. Of the remaining municipalities, 22.4% (n = 191) were classified as high-high, 30.0% (n = 256) as low-low, and few municipalities were located in the low-high (n = 15; 1.8%) and high-low (n = 16; 1.9%) quadrants (Fig 5).

The macroregions with the highest number of municipalities with high rates, surrounded by other municipalities also with high rates (high-high), were Northwest (100%), East (91.7%), South East (83.3%), North (81.5%) and Northeast (77.6%). The macroregions that presented low-low risk were Southeast (97.9%), Center (85.9%), South Center (85.1%), South (61.8%) and Jequitinhonha/Diamantina (52.4%).

## Discussion

Notification of new leprosy cases in the general population and in the 0 to 14 aged group decreased in Minas Gerais from 2004 to 2019. However, despite this reduction, both rates remain above the parameters recommended by the Brazilian MS, classified as medium endemicity [9].

The same can be observed with the grade 2 physical disability rate at diagnosis. Despite presenting more significant oscillations over the years, a reduction was also observed during the study period. It is noteworthy that the analysis of this indicator should be used in conjunction with the general detection rate and in children under 15 years old to monitor the trend of timely detection and disease control activities, in addition to measuring the need for physical and social rehabilitation of the disease [9,21].

The evident decreasing trend of the epidemiological indicators studied is consistent with the Brazilian trend, which can be attributed to leprosy control actions, with the implementation of multidrug therapy, the municipalization of health services and the increase in coverage of the Family Health Strategy (FHS) over the years [22,23], which strengthened the disease control actions [24,25].

However, the stability of the proportion of grade 2 physical disability at high levels shows late diagnosis and contributes to the formation of transmission foci [7,26]. In addition, a recent study that estimated the underreporting of leprosy cases by the proportion of grade 2 physical disability conducted throughout the national territory highlighted the state of Minas Gerais as one of the regions with a lower probability of notifying leprosy and which registered fewer new cases [26].

Thus, despite the increased FHS coverage and advances in primary health care being relevant to the fall of the endemic disease, surveillance actions should be active, and health professionals trained to recognize the signs and symptoms of the disease and enable early diagnosis [27,28]. Even in regions of low endemicity, where the disease is stable, there may be active transmission [29].

Besides the actions of health services being relevant for leprosy control, it is already known that the most unfavorable socioeconomic and demographic conditions have a historical relationship with the epidemiology of infectious diseases [2,30]. Furthermore, although the present study did not directly analyze the relationship between socioeconomic inequalities and leprosy epidemiological risk, its spatial concentration and persistence in some geographical regions have been correlated both in studies involving large areas, such as states [7,18,31] and national [5] and in small areas, such as municipalities and census tracts [29,32,33].

In this study, in both study periods, the 213 municipalities classified as having high epidemiological risk were distributed throughout the state, with municipalities with high rates being close to municipalities with lower rates. The largest concentration of these municipalities is located in the Northeast, Northwest, and South East macroregions. We also highlight the North region, which increased the concentration of municipalities with medium and high epidemiological risk if we compare the two periods analyzed. The formation of clusters of epidemiological risk was confirmed with Moran’s Global Index, which indicates that the analyzed data set is grouped.

The autocorrelation analysis confirmed this distribution, in which a higher percentage of municipalities were classified as "high-high" in the macroregions Northwest, East, Northeast, South East and West from 2004 to 2011. Moreover, from 2012 to 2019, the Northwest, East, South East and Northeast macroregions remained with a higher concentration of municipalities classified as "high-high", and the North macroregion became more expressive than in the first period.

Thus, with the use of spatial analysis, it was possible to verify that, despite the leprosy decline in the state of Minas Gerais, the macroregions Northwest, Northeast, East, and South East stand out, with a higher proportion of municipalities identified as a high epidemiological risk throughout the study period, and also the macroregion North that gained epidemiological importance in the last period analyzed. This corroborates previous research on the increase in high risk in regions with more significant social inequalities.

The most critical areas pointed out in the study have already been highlighted in the literature as historically endemic regions. A study conducted in the Jequitinhonha Valley, located in the Northeast macroregion of the state, from 1998 to 2006, warned about the region’s late diagnosis [34]. This points to the need to intensify active search actions in areas with a history of endemicity, suggesting that a hidden prevalence contributes to the disease transmission chain.

In addition, the Northeast macroregion still had a silent municipality (Monte Formoso), which did not notify any case of the disease, inserted in one of the risk clusters to detect the disease in the state [5,35]. It is known that municipalities with very high rates are usually neighbors with others with high or intermediate rates. Thus, intensive monitoring is crucial to institute more effective measures to control the disease [36].

The municipalities included in the low leprosy epidemiological risk cluster should be highlighted and analyzed with caution because these municipalities may actually be free of the disease or, on the other hand, it may be an underreporting of cases or a populous municipality in which the number of cases is diluted and concentrated in specific areas. A study in the state capital, Belo Horizonte, inserted in the center macroregion, pointed to the concentration of cases in the urban peripheries, relating it to the most vulnerable populations [33].

Thus, we emphasize the need for greater detailing of the areas with the highest leprosy epidemiological risk, analyzing the territory in spatial units with a higher level of disaggregation, and understanding the conditions and determinants factors in the maintenance of the disease in the state’s municipalities. The limitations of this study were the use of secondary data sources, which may present inconsistencies in the quality and quantity of information. However, the data were treated with methodological rigor and represented the epidemiological situation of leprosy in the studied period, not making the analysis unfeasible. Emphasizing its importance for monitoring and evaluating the epidemiological situation of leprosy and for planning health surveillance actions.

## Conclusions

Leprosy has a heterogeneous spatial pattern and remains concentrated in historically endemic areas of the state. It is concentrated in Northwest, Northeast, North, East, and South East macroregions. The use of spatial analysis contributed to identifying areas of higher leprosy epidemiological risk in the state of Minas Gerais. Such information would not be visualized by working only with tabular data.

Our results provide potential pathways to target leprosy control programs in the decision-making of health professionals and managers about health surveillance and epidemiological surveillance. Especially in areas identified as having higher epidemiological risk, and enable the formulation of public policies for disease control, considering the different epidemiological scenarios present in the state.

It reinforces the importance of intensifying actions to combat leprosy in these municipalities and macroregions, especially those aimed at early detection of cases, examining the community in schoolchildren, and including interventions that reduce social inequalities. Promoting improved access to health services and combating stigma and prejudice to eliminate leprosy as a public health problem.

## References

[1] World Health Organization. Global leprosy update, 2015: time for action, accountability and inclusion. Wkly Epidemiol Rec. 2016,9(35):405–20. 27592500

[2] Pescarini JM, Strina A, Nery JS, Skalinski LM, Andrade KVFD, Penna MLF, Brickley EB, Rodrigues LC, Barreto ML, Penna GO. Socioeconomic risk markers of leprosy in high-burden countries: A systematic review and meta-analysis. PLoS Negl Trop Dis. 2018,12(7):e0006622. doi: 10.1371/journal.pntd.0006622 29985930 PMC6053250

[3] World Health Organization. Global leprosy update, 2016: accelerating reduction of disease burden. Wkly Epidemiol Rec. 2017,92(35):501–19. 28861986

[4] World Health Organization. Global leprosy (Hansen disease) update, 2019: time to step-up prevention initiatives. Wkly Epidemiol Rec. 2020,95(36):417–40.

[5] Rodrigues RN, Leano HAM, Bueno IC, Araújo KMFA, Lana FCF. High-risk areas of leprosy in Brazil between 2001–2015. Rev Bras Enferm. 2020,73(3):1–7. doi: 10.1590/0034-7167-2018-0583 32294707

[6] Freitas LRS, Duarte EC, Garcia LP. Trends of main indicators of leprosy in Brazilian municipalities with high risk of leprosy transmission, 2001–2012. BMC Infect Dis. 2016,16(472). doi: 10.1186/s12879-016-1798-2 27595751 PMC5011946

[7] Pereira KC, Bueno IC, Lana FCF. Tendência epidemiológica da hanseníase em Minas Gerais (1995–2015). Cogitare Enferm. 2019,24:e66109. doi: 10.5380/ce.v24i0.66109 Portuguese.

[8] Gerais Minas. Secretaria de Estado da Saúde. Plano Estadual de Enfrentamento da Hanseníase. Belo Horizonte: UFMG; 2019. Portuguese.

[9] Brasil. Ministério da Saúde. Departamento de Vigilância das Doenças Transmissíveis. Diretrizes para vigilância, atenção e eliminação da hanseníase com problema de saúde pública: manual técnico-operacional. Brasília-DF: Ministério da Saúde; 2016. Portuguese.

[10] Brasil. Ministério da Saúde. Guia de Vigilância em Saúde. 3rd ed. Brasília-DF: Ministério da Saúde; 2019. Portuguese.

[11] Silva CLM, Fonseca SC, Kawa H, Palmer DOQ. Spatial distribution of leprosy in Brazil: a literature review. Rev Soc Bras Med Trop. 2017,50(4):439–49. doi: 10.1590/0037-8682-0170-2016 28954063

[12] Druck S, Carvalho MS, Câmara G, Monteiro AMV. Análise espacial de dados geográficos. Brasília: EMBRAPA; 2004. Portuguese.

[13] Brasil. Ministério da Saúde. Vigilância em Saúde: situação epidemiológica da hanseníase no Brasil. Brasília-DF: Ministério da Saúde; 2008. Portuguese.

[14] Nardi SMT, Paschoal JAA, Pedro HSP, Paschoal VD, Sichieri EP. Geoprocessamento em Saúde Pública: fundamentos e aplicações. Rev Inst Adolfo Lutz. 2013,72(3):185–91. doi: 10.18241/0073-98552013721562 Portuguese.

[15] Organización Panamericana de la Salud. Uso de los sistemas de información geográfica en epidemiología (SIG-Epi). Boletín Epidemiológico. 1996,17(1). Portuguese.

[16] Ramos AC, Yamamura M, Arroyo LH, Popolin MP, Chiaravalloti Neto F, Palha PF, Uchoa SA, Pieri FM, Pinto IC, Fiorati RC, Queiroz AAR, Belchior AS, Santos DT, Garcia MC, Crispim JA, Alves LS, Berra TZ, Arcêncio RA. Spatial clustering and local risk of leprosy in São Paulo, Brazil. PLoS Negl Trop Dis. 2017,11(2):e0005381. doi: 10.1371/journal.pntd.0005381 28241038 PMC5344525

[17] Ignotti E, Paula RC. Situação epidemiológica da hanseníase no Brasil: análise de indicadores selecionados no período de 2001 a 2010. In: Brasil. Ministério da Saúde. Saúde Brasil 2010: uma análise da situação de saúde e de evidências selecionadas de impacto de ações de vigilância em saúde. Brasília-DF: Ministério da Saúde; 2011, Portuguese.

[18] Araújo KMFA, Gomes LCF, Lana FCF. Spatial analysis of the risk of leprosy disease in a northeastern brazilian state. Rev baiana enferm. 2020,34:e37902. doi: 10.18471/rbe.v34.37902

[19] Assunção RM, Barreto SM, Guerra HL, Sakurai E. Mapas de taxas epidemiológicas: uma abordagem Bayesiana. Cad Saude Publica. 1998,14(4):713–23. doi: 10.1590/S0102-311X1998000400013 Portuguese. 9878904

[20] Brasil. Ministério da Saúde. Fundação Oswaldo Cruz. Introdução à Estatística Espacial para a Saúde Pública. Brasília-DF: Ministério da Saúde, 2007. Portuguese.

[21] Declercq E. Reflections on the new WHO leprosy indicator: The rate of new cases with grade 2 disabilities per 100,000 population per year. Leprosy Review. 2011,82(1):3–5. 21644466

[22] Gomes FBFF, Lana FC, Oliveira RC, Rodrigues RN. Indicators of Leprosy in the State of Minas Gerais and Its Relationship With the Municipal Human Development Index and the Coverage of the Family Health Strategy. Rev Min Enferm. 2017,21:e-1063. doi: 10.5935/1415-2762.20170073

[23] Monteiro LD, Martins-Melo FR, Brito AL, Lima MS, Alencar CH, Heukelbach J. Tendências da hanseníase no Tocantins, um estado hiperendêmico do Norte do Brasil, 2001–2012. Cad. saúde pública. 2015,31(5):971–80. doi: 10.1590/0102-311X00075314 Portuguese. 26083172

[24] Brasil. Portaria no 1838, de 09 de outubro de 2002. Brasília: Diário Oficial da República Federativa do Brasil, 2002. Portuguese.

[25] Dias RC, Pedrazzani ES. Políticas públicas na Hanseníase: contribuição na redução da exclusão social. Rev. bras. enferm. 2008,61 Spec:753–6. doi: 10.1590/S0034-71672008000700016 Portuguese. 19009119

[26] Oliveira GL, Oliveira JF, Pescarini JM, Andrade RFS, Nery JS, Ichihara MY, Brickley EB, Barreto ML, Penna GO, Penna MLF, Sanchez M. Estimating underreporting of leprosy in Brazil using a Bayesian approach. PLoS Negl Trop Dis. 2021,15(8):e0009700. doi: 10.1371/journal.pntd.0009700 34432805 PMC8423270

[27] Smith CS, Noordeen SK, Richardus JH, Sansarricq H, Cole ST, Soares RC, Savioli L, Aerts A, Baruaf S. A strategy to halt leprosy transmission. Lancet Infect Dis. 2014,14(2):96–8. doi: 10.1016/S1473-3099(13)70365-7 24457165

[28] Vieira NF, Lanza FM, Martínez-Riera JR, Nolasco A, Lana FCF. Orientación de la atención primaria en las acciones contra la lepra: factores relacionados con los profesionales. Gaceta Sanitaria. 2020,34(2):120–6. doi: 10.1016/j.gaceta.2019.02.011 Spanish. 31053453

[29] Ramos ACV, Martoreli Júnior JF, Berra TZ, Alves YM, Barbosa TP, Scholze AR, Assis IS, Palha PF, Gomes D, Arcêncio RA. Temporal evolution and spatial distribution of leprosy in a municipality with low endemicity in São Paulo state, Brazil. Epidemiol. Serv. Saúde. 2022,31(1):e2021951. doi: 10.1590/S1679-49742022000100018 35476004

[30] Leano HAM, Araújo KMFA, Rodrigues RN, Bueno IC, Lana FCF. Indicators related to physical disability and diagnosis of leprosy. Rev Rene. 2017,18(6):832–9. doi: 10.15253/2175-6783.2017000600018

[31] Souza CDF, Luna CF, Magalhães MAFM. Spatial modeling of leprosy in the state of Bahia and its social determinants: a study of health inequities. Anais Brasileiros de Dermatologia. 2019,94(2):182–91. doi: 10.1590/abd1806-4841.20197554 31090823 PMC6486086

[32] Barreto JG, Bisanzio D, Guimarães LS, Spencer JS, Vazquez-Prokopec GM, Kitron U, Salgado CG. Spatial Analysis Spotlighting Early Childhood Leprosy Transmission in a Hyperendemic Municipality of the Brazilian Amazon Region. PLoS Negl Trop Dis. 2014,8(2)e2665. doi: 10.1371/journal.pntd.0002665 24516679 PMC3916250

[33] Rodrigues RN, Niitsuma ENA, Bueno IC, Baquero OS, Jardim CCG, Lana FCF. Leprosy and health vulnerability in Belo Horizonte, Minas Gerais. Rev Min Enferm. 2017,21:e-997. doi: 10.5935/1415-2762.20170007

[34] Lana FCF, Amaral EP, Lanza FM, Saldanha ANSL. Physical disabilities resulting from hansen’s disease in Vale do Jequitinhonha/state of Minas Gerais, Brazil. Rev Latino-Am Enfermagem. 2008,16(6):993–7. doi: 10.1590/s0104-11692008000600009 19229402

[35] Amaral EP, Lana FCF. Spacial analysis of Leprosy in the microregion of Almenara, MG, Brazil. Rev bras enferm. 2008,61 Spec:701–7. doi: 10.1590/s0034-71672008000700008 19009111

[36] Souza EA, Ferreira AF, Heukelbach J, Boigny RN, Alencar CH, Ramos-Júnior AN. Epidemiology and spatiotemporal patterns of leprosy detection in the State of Bahia, Brazilian Northeast Region, 2001–2014. Trop Med Infect Dis. 2018,3(3):79. doi: 10.3390/tropicalmed3030079 30274475 PMC6161284

